# Strategies to overcome resistance to enfortumab vedotin and pembrolizumab for patients with urothelial carcinoma: harnessing present knowledge for future advances

**DOI:** 10.37349/etat.2025.1002307

**Published:** 2025-04-07

**Authors:** Albert Jang, Jason R. Brown

**Affiliations:** IRCCS Istituto Romagnolo per lo Studio dei Tumori (IRST) “Dino Amadori”, Italy; ^1^Division of Solid Tumor Oncology, Department of Medicine, University Hospitals Cleveland Medical Center, Case Western Reserve University School of Medicine, Cleveland, OH 44106, USA; ^2^Case Comprehensive Cancer Center, Cleveland, OH 44106, USA

**Keywords:** Primary resistance, secondary resistance, enfortumab vedotin, pembrolizumab, urothelial carcinoma

## Abstract

The combination of enfortumab vedotin and pembrolizumab (EVP) has been recently approved for patients with locally advanced and metastatic urothelial carcinoma. This combination showed a higher objective response rate and superior progression-free survival and overall survival over traditional platinum-based chemotherapy in the frontline setting in the pivotal EV-302 trial. Despite the success, a subset of patients has primary refractory disease, and another subset will develop secondary resistance over time. Resistance to enfortumab vedotin may include the downregulation of nectin-4 expression to minimize antibody binding, upregulation of efflux pumps against the toxin, or direct resistance by the tubulin against the toxin. Resistance to pembrolizumab includes several methods to downregulate the immune system. Additionally, the type of histology of the urothelial carcinoma likely plays an important role in resisting EVP. This review summarizes these possible mechanisms of primary and secondary resistance, potential biomarkers predictive of response and resistance, and methods to overcome the resistance to EVP.

## Introduction

Historically, platinum-based chemotherapy (PBC) has been the backbone of systemic therapy for patients with advanced urothelial carcinoma (aUC), and there were limited options afterwards [1]. Significant advances have occurred in the past decade especially with the use of immune checkpoint inhibitors (ICIs) and antibody drug conjugates (ADCs). Enfortumab vedotin (EV), an ADC linking monomethyl auristatin E (MMAE) toxin to an antibody targeting nectin-4, was initially approved as a third-line regimen after progression on PBC and ICI [2].

The combination of EV and pembrolizumab (EVP) was initially evaluated as frontline therapy for cisplatin-ineligible patients with aUC in the phase 1/2 EV-103 trial [3]. The promising results led to the phase 3 EV-302 trial evaluating EVP versus PBC for patients with aUC regardless of cisplatin eligibility. This trial demonstrated a median overall survival (mOS) of 31.5 months for EVP versus 16.1 months for PBC [4], shifting the treatment paradigm dramatically as a new standard of care for patients with aUC. Despite the overwhelming success of EVP, several issues remain.

Cancer resistance can be categorized into either having primary refractory disease when tumors continue to enlarge and metastasize despite the initiation of treatment, compared to secondary resistance (relapsed disease) where tumors initially are stable or regress in size for some time before enlarging and metastasizing. Almost 10% of patients receiving EVP in EV-302 had progressive disease as best objective response, suggesting primary refractory disease. An additional 50% of patients progress on EVP by two years, suggesting secondary resistance. Because of the novelty of EVP as a frontline regimen, there is currently no standard second line therapy, although potential considerations include PBC, or erdafitinib if there is an *FGFR2/3* alteration [5]. Understanding the mechanisms of resistance to EVP would provide insights into designing new therapies and could lead to more knowledge in better sequencing of subsequent treatments.

To better understand resistance, it is essential to consider the molecular and epigenetic underpinnings of the development and progression of urothelial carcinoma. Various studies at the genomic, transcriptional, and cellular levels, show both intratumoral and intertumoral heterogeneity play a role in cancer growth, suggesting that over time, single-agent therapy is unlikely to be successful in controlling aUC [6]. Using The Cancer Genome Atlas (TCGA), a comprehensive analysis of 412 muscle-invasive bladder cancers showed several significantly mutated genes and several other genes subject to epigenetic silencing, as well as overall five expression-based subtypes [7]. This heterogeneity makes it a challenge to design therapies that can minimize the development of resistance.

**Figure 1:**
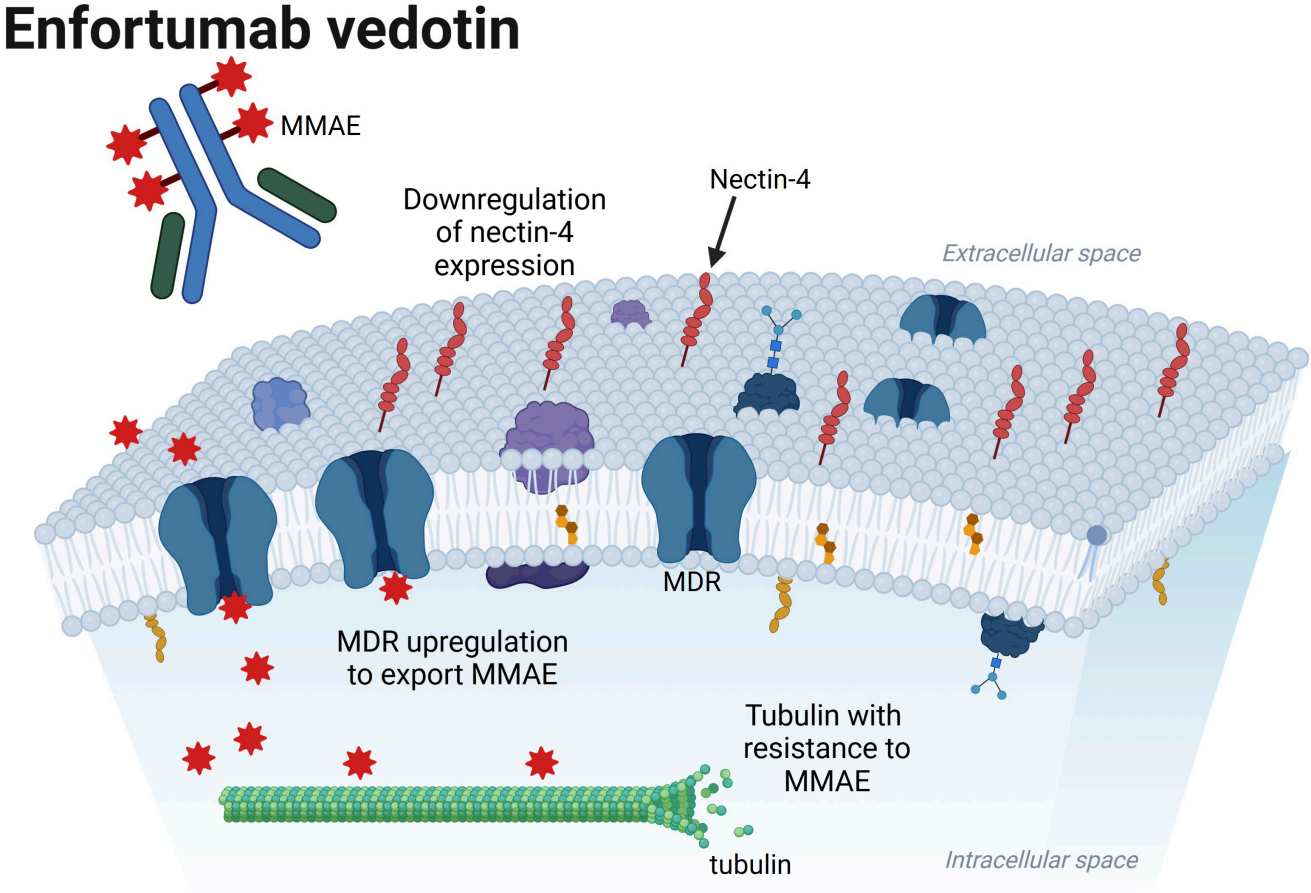
**Possible mechanisms of resistance to enfortumab vedotin in the urothelial cancer cell, which include downregulation of nectin-4 expression, multi-drug resistance (MDR) upregulation to export MMAE, and tubulin directly resistant to MMAE**. MMAE: monomethyl auristatin E. Created in BioRender. Jang, A. (2025) https://BioRender.com/w62e363

**Figure 2:**
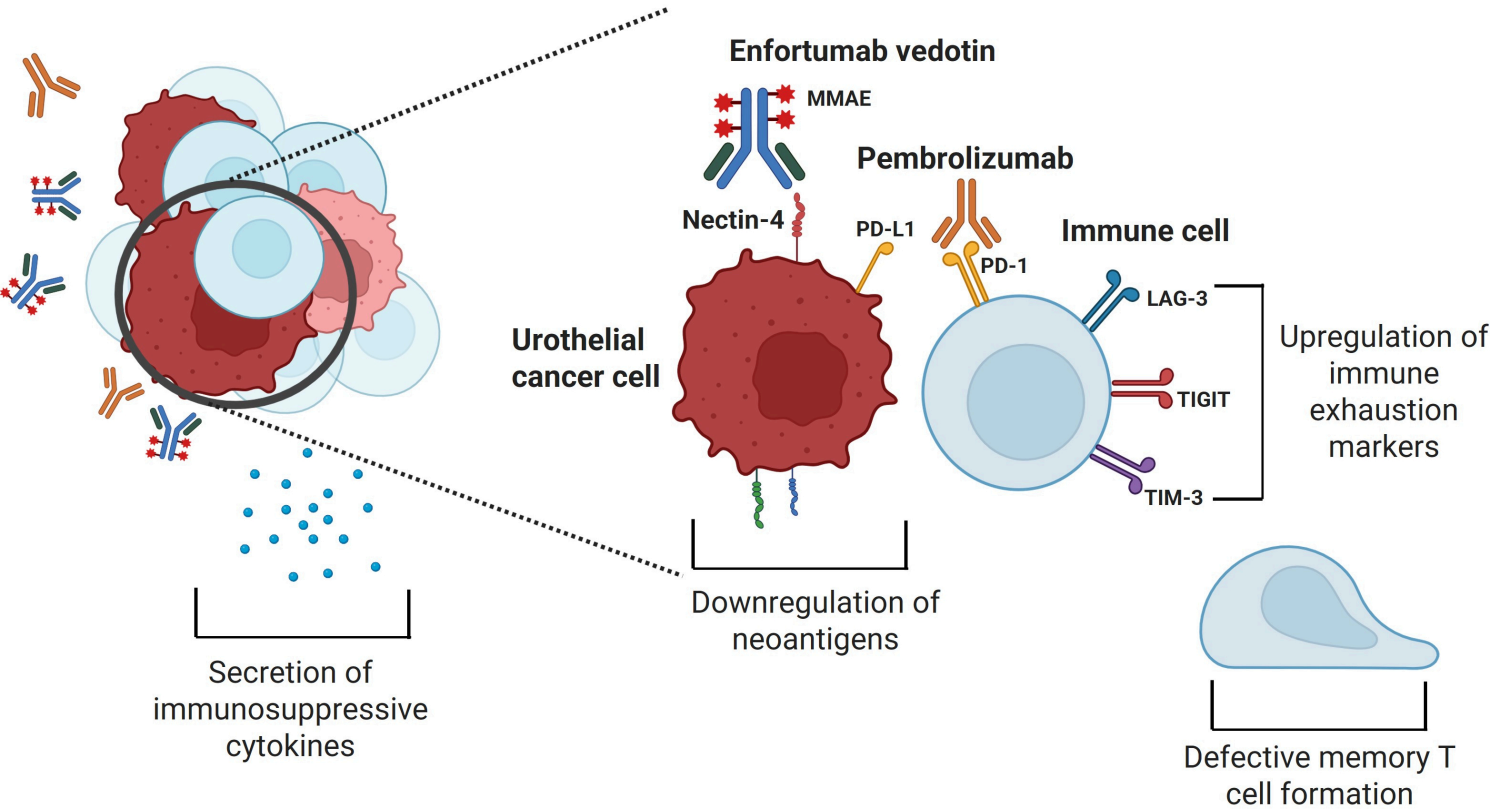
**Possible mechanisms of resistance to pembrolizumab, which include upregulation of immune exhaustion markers, immunosuppressive cytokine release, downregulation of neoantigens by tumor cells, and defective formation of memory T cells**. MMAE: monomethyl auristatin E; PD-L1: programmed cell death protein ligand 1; PD-1: programmed cell death protein 1; LAG-3: lymphocyte activation gene-3; TIGIT: T cell immunoreceptor with immunoglobulin and ITIM domain; TIM-3: T-cell immunoglobulin and mucin domain-containing molecule 3. Created in BioRender. Jang, A. (2025) https://BioRender.com/w62e363

The general mechanisms of resistance that cancer cells possess against ADCs can be broadly grouped into the following areas: cell surface antigen expression, ADC processing, and drug payload. For EV, this corresponds to changes in the tumor cell surface expression of nectin-4, processing of the protease-cleavable linker and MMAE in tumor cells, and resistance to the MMAE itself (Figure 1). For pembrolizumab, resistance includes upregulation of an immunosuppressive environment and immune exhaustion (Figure 2). In this review, we discuss the current knowledge of the mechanisms of resistance to EVP for aUC.

## Molecular features of resistance

### Nectin-4

Nectin-4 is an immunoglobulin-like transmembrane protein important for cell-cell adhesion [8] and is almost ubiquitously expressed in UC, making it a desirable target. The intensity of nectin-4 expression can be characterized with the immunohistochemical (IHC) scoring (H-score) system, which is the sum of the staining intensity characterized by pathology (0–3) multiplied by the percentage of cells (0–100) stained at the given intensity, resulting in a range of 0–300. In an IHC analysis of a wide range of solid tumor specimens, there were 524 bladder cancer specimens analyzed for nectin-4 expression, with 31% having strong intensity (H-score 200–300), 29% having moderate intensity (H-score 100–199), 23% with weak intensity (H-score 15–99), and only 17% of samples staining negative (H-score 0–14) [9]. Similarly, nectin-4 expression for 99 samples of upper tract UC revealed 10.1% with strong intensity, 24.2% with moderate intensity, and 31.3% with weak intensity, with 34.3% of samples with negative staining [10]. In the EV-101 phase 1 trial, which for the first time evaluated the safety, tolerability, and antitumor activity of EV for patients with aUC, a documented nectin-4 H-score was initially required prior to enrollment [11]. The median H-score for the 152 tested tumor samples was 290, with only five samples having an H-score < 150 including one sample with H-score 0. Because of the overwhelming positivity, nectin-4 scoring was not required for the EV trials afterwards.

The surface expression of nectin-4 may differ between primary UC and metastatic sites, potentially driving resistance. In a recent analysis, 137 patient samples of primary tumor with matched synchronous or metachronous metastases were stained [12]. For primary tumor specimens, median H-score was 110 (interquartile range 25–200), including 19.7% negative samples. For corresponding metastatic specimens, median H-score was 40 (interquartile range 0–140) with 39.4% negative samples. Decrease of nectin-4 expression with metastatic spread was statistically significant (*P* < 0.001). In this analysis, 47 patients received EV, and absent or weak nectin-4 staining was associated with significantly worse progression-free survival (PFS) compared to moderate/strong staining. High nectin-4 expression also significantly correlated with radiographic partial or complete response.

Therefore, nectin-4 expression is a potential predictive marker of response to EV. Tumors with absent or weak expression of nectin-4 in primary or metastatic tissue may be associated with primary refractory disease, and downregulation of nectin-4 expression with treatment could mediate secondary resistance. Because EV binds selectively to nectin-4, EV intuitively may bind tumor cell surfaces that overexpress nectin-4 more effectively compared to cell surfaces that underexpress or do not express nectin-4.

This principle was evaluated in an exploratory analysis of nectin-4 expression using immunohistochemistry assay of primary or metastatic tissue from patients enrolled in EV-302 [13]. For 800 available samples, the median H-score was 275, with a median of 280 (interquartile range 230–298) specifically for the EVP arm. In the EVP arm, only 9.6% of samples exhibited H-score < 150. Patients on the EVP arm with H-score < 275 had median PFS (mPFS) of 10.4 months (95% CI, 8.2–14.5) and mOS of 26.1 months (95% CI, 22.3–NR), compared to mPFS of 15.3 months and mOS of NR (95% CI, 25.6–NR) for patients with H-score ≥ 275. Objective response rates were 52.6% for H-score < 150, 58.0% for H-score ≥ 150 to < 225, and 71.8% for H-score ≥ 225. An exploratory analysis evaluating patients with available nectin-4 expression levels (H-score ≥ 275 or < 275) and programmed cell death protein ligand 1 (PD-L1) expression [based on the combined positive score (CPS), with < 10 considered low and ≥ 10 considered high] is summarized in Table 1. All subsets were significantly better for EVP compared to the PBC control arm (mPFS of 6.2–6.4 months with little variation), regardless of nectin-4 expression and PD-L1 status. This analysis did suggest there were better outcomes for patients on EVP with higher levels of nectin-4 expression.

**Table 1:** The mPFS and mOS values for patients in EV-302 trial with available H-score and PD-L1 CPS values

**H-score and PD-L1 CPS**	**mPFS**	**mOS**
H-score < 275 (irrespective of PD-L1)	10.4 months (95% CI, 8.2–14.5)	26.1 months (95% CI, 22.3–NR)
H-score ≥ 275 (irrespective of PD-L1)	15.3 months (95% CI, 10.5–NR)	NR (95% CI, 25.6–NR)
H-score < 275 and PD-L1 CPS < 10	9.2 months (95% CI, 6.2–12.0)	22.3 months (95% CI, 17.0–NR)
H-score < 275 and PD-L1 CPS ≥ 10	10.6 months (95% CI, 8.1–NR)	31.5 months (95% CI, 25.4–NR)
H-score ≥ 275 and PD-L1 CPS < 10	11.6 months (95% CI, 8.2–22.3)	NR (95% CI, NR–NR)
H-score ≥ 275 and PD-L1 CPS ≥ 10	20.4 months (95% CI, 12.0–NR)	25.6 months (95% CI, 21.2–NR)

CPS: combined positive score; mPFS: median progression-free survival; mOS: median overall survival; PD-L1: programmed cell death protein ligand 1

With exposure to EV, secondary resistance may occur with downregulation of nectin-4 expression. In a case report of one patient who developed resistance to EV, IHC analysis from metastasectomy of two enlarging oligometastatic disease sites in the lung and adrenal gland were analyzed. There was decreased expression of nectin-4 in these metastatic sites compared to their expression on the primary tumor [14]. Nectin-4 scoring over time in the development of resistance to EV from large cohorts is lacking, but there is data from other approved ADCs regarding downregulation of the surface antigen. In the DAISY trial of patients who received trastuzumab deruxtecan (T-DXd), an ADC that binds to HER2, an analysis of 20 patients who developed secondary resistance showed 13 patients had a decrease in HER2 immunohistochemistry expression [15].

### Nectin-4 expression in histologic subtypes

UC tumors of subtype histologies have generally been excluded from clinical trials. These tumors tend to be more aggressive and have a worse prognosis [16]. There has been recent evidence that nectin-4 expression is lower in these histologies, which likely has important implications for the effectiveness of EV.

One study characterized nectin-4 expression using IHC from radical cystectomy samples among the three most frequent variant subtypes: squamous cell carcinoma, adenocarcinoma, and sarcomatoid UC [17]. The median H-score of nectin-4 was the lowest for sarcomatoid UC (10, range 0–185), compared to squamous cell carcinoma (150, range 0–250), and adenocarcinoma (140.5, range 30–275).

Another study evaluated nectin-4 expression of 169 patients on several different histologic subtypes [18]. Subtypes with low rates of nectin-4 positivity included sarcomatoid carcinoma (10% of available samples), small cell carcinoma (0%), and micropapillary tumors (28%), all aggressive subtypes. Therefore, low nectin-4 expression may reduce EV efficacy for tumors with a significant proportion of these rare subtypes.

In the Urothelial Cancer Network to Investigate Therapeutic Experiences (UNITE) database, the efficacy of EV monotherapy was assessed in a real-world setting for 566 patients with documented histology (366 with pure UC, 200 with either pure variant or component of variant histologies) [19]. Patients with predominant variant histology (> 50%) had a numerically lower observed response rate (ORR) compared to patients with pure UC, but there were no differences in mPFS or mOS. Notably, patients with pure variant histology had worse outcomes compared to patients with pure UC in terms of ORR (0% versus 52%, *P* < 0.001), mPFS (HR 1.9, 95% CI 1.06–3.39, *P* = 0.03), and mOS (HR 2.96, 95% CI 1.160–5.46, *P* < 0.001). These hypothesis-generating results suggest that majority or pure subtype histologies may be associated with primary refractory disease to EV. Moreover, there may be implications in terms of primary or secondary resistance for tumors with predominant urothelial histology and minority subtype histology.

### MMAE resistance

MMAE, a synthetic derivative of dolastatin 10, is an antimitotic agent that inhibits the polymerization of tubulin, leading to microtubule dysregulation [20]. Although the mechanisms of resistance to MMAE have not been fully elucidated, they may be extrapolated from vinca alkaloids and taxanes, which are also disruptors of microtubules. Resistance to these agents is diverse and can include the upregulation of efflux pumps by the cancer cell, the upregulation of vaults which are cytoplasmic organelles that trap cytotoxic drug to prevent their ability to bind to its target, the upregulation of detoxification enzymes to break down the toxin, and the upregulation of tubulin synthesis or the development of mutations to minimize interaction with the toxin [21, 22]. If there is resistance to MMAE from EV, a subsequent vedotin-based treatment, such as disitamab vedotin (DV), would be less efficacious.

### EV processing

Cancer cells not only can directly become resistant to chemotherapy intracellularly, but studies have also shown they can actively pump out the intracellular toxins. ATP-binding cassette transporters such as multi-drug resistance 1 (MDR1), also known as P-glycoprotein, have been well characterized to pump toxins like MMAE out of cells. These transporters can be upregulated in chemoresistant cancer cells [23]. In a preclinical UC model using the RT112 cell line, secondary resistance to EV was noted to be associated with an increase in *MDR1* gene expression and *TGF-β* gene expression, without much downregulation of nectin-4 [24]. In the case report of one patient who developed resistance to EV as described above, IHC analysis from metastasectomy of two enlarging oligometastatic disease sites in the lung and adrenal gland were analyzed. There was decreased expression of nectin-4 and increased expression of MDR1 in these metastatic sites compared to their expression on the primary tumor, respectively [14]. Like EV, brentuximab vedotin (BV), which is an ADC that binds to CD30, also uses MMAE as its toxin. The mechanisms of resistance for BV against MMAE include increased expression of MDR1, of which MMAE is a substrate that leads to its efflux out of cancer cells [25, 26].

The inhibition of these drug efflux pumps appears to be effective in resensitizing cancer cells to the original chemotherapy agent. The MDR1 protein inhibitor tariquidar was demonstrated in a murine model with ADC-resistant tumors to safely reverse the ADC-induced resistance [27]. Cyclosporine, which competitively inhibits MDR1 [28], has been evaluated in a phase 1 trial (NCT03013933) in combination with BV for patients with relapsed/refractory Hodgkin lymphoma. In this cohort already refractory to BV, the reported overall response rate was 75% [29]. Therefore, MDR1 inhibitors may be useful for patients whose tumors develop resistance to EV over time, which must be validated prospectively.

## Genetic alterations as predictive markers of response

### *NECTIN4* gene amplification

Another way of quantifying nectin-4 expression is characterization of the *NECTIN4* gene using either DNA or mRNA expression, which may be a more objective method of measurement compared to the H-score. The *NECTIN4* mRNA expression was measured within muscle-invasive UC specimens in one study [30]. This study found heterogeneous *NECTIN4* expression between molecular subtypes, with significant expression in luminal subtypes and lower expression in basal, neuroendocrine-like, and stroma-rich subtypes. Moreover, knockdown of *NECTIN4* in luminal cells led to EV resistance, while upregulation of *NECTIN4* in basal cells led to increased EV sensitivity. This suggests the importance of *NECTIN4* gene expression predicting treatment response to EV.

In clinical practice, from a cohort of 108 patients with aUC with available tumor tissue samples and treated with EV, *NECTIN4* copy number variants were assessed via fluorescence in situ hybridization (FISH) [31]. *NECTIN4* amplifications were detected in 26% (28/108) of samples. The *NECTIN4*-amplified tumors correlated with higher nectin-4 expression than *NECTIN4* non-amplified tumors (median H-score 295 versus 90, interquartile range 235–300 versus 20–205). Among 27 available matched primary and metastatic samples, the *NECTIN4* copy number variation was stable in 93% of samples, with 7 of 8 *NECTIN4-*amplified primary tumor tissue samples retaining the amplification in the matched metastatic sample. For the 28 patients with *NECTIN4* amplification, the objective response rate on EV was 96%, compared to just 32% for the 80 patients without *NECTIN4* amplification. In contrast to changes in nectin-4 H-score between primary and metastatic disease, low *NECTIN4* amplification in the primary specimen can be potentially used to predict primary resistance in aUC. The importance of *NECTIN4* amplification of the tumor tissue and its association with EV response should be further investigated. Additionally, there may be downregulation of *NECTIN4* with EV exposure over time, so this may be a method to monitor secondary resistance.

Next-generation sequencing (NGS) to evaluate specific gene alterations associated with EV resistance has been assessed retrospectively. In the UNITE database, 124 of 488 patients (25.4%) had primary refractory disease, defined as progressive disease as best response to EV monotherapy [32]. For patients with available NGS data, it was noted that patients with primary refractory disease had a higher rate of *CDKN2B* alterations (19% versus 12%, *P* = 0.05). Another analysis of the UNITE database identified alterations in *ERBB2* and *KDM6A*, as well as high tumor mutational burden, as biomarkers predictive of better overall survival (OS) in patients who received EV [33]. A small single-institution study of 29 patients with aUC receiving EV found *TP53* and *MDM2* alterations were predictive of higher ORR, longer PFS, and longer OS [34]. The significance of these genomic alterations for EV resistance would have to be assessed in prospective studies.

Genomic alterations have also been implicated in resistance to EVP. In analysis of 118 patients treated with EVP in the UNITE database, *TP53* and *KMT2D* alterations were associated with worse outcomes on EVP [35]. In multivariate analysis including age, race, histology, clinical performance status, blood counts, and albumin as covariates, patients had worse PFS with *KMT2D* alterations (HR 2.2; 95% CI, 1.0–4.5; *P* = 0.04) and *TP53* alterations (HR 2.3; 95% CI, 1.2–4.2; *P* < 0.01], and worse OS with *TP53* alterations (HR 2.3; 95% CI, 1.0–5.4, *P* = 0.05]. How these gene alterations contribute to treatment resistance of EVP needs further investigation.

## Immunologic features of resistance

The complex mechanisms of resistance to ICIs have been extensively reviewed in the literature [36–39]. Primary resistance to ICIs can be due to downregulation of the immune system by various mechanisms including tumor-intrinsic factors such as alterations in neoantigen formation, processing, and presentation and tumor-extrinsic factors including a disrupted functional ability of antigen-presenting cells and cytotoxic T cells due to an unfavorable tumor microenvironment. Cancer cells can develop secondary resistance to ICIs through immune exhaustion, which includes the upregulation of different immune checkpoints on cytotoxic T cells with chronic antigen exposure including lymphocyte activation gene-3 (LAG-3), T cell immunoreceptor with immunoglobulin and ITIM domain (TIGIT), and T-cell immunoglobulin and mucin domain-containing molecule 3 (TIM-3), secretion of immunosuppressive cytokines, tumor neoantigen downregulation, and a defective formation of memory T cells.

For aUC, ICI monotherapy only has an objective response rate of 20–30%, with a large proportion of patients having primary refractory disease [40–42]. Notably, nectin-4 has recently been found to be a functional ligand for TIGIT. In preclinical studies, the nectin-4 and TIGIT interaction inhibited natural killer cell activity, and nectin-4 blocking antibodies enhanced tumor cell killing [43]. This may be one possible mechanism for the resistance to ICI monotherapy of UC tumors. Identifying other reasons to how UC tumors can become resistant to ICIs is underway.

One study characterized features associated with ICI resistance in aUC using bulk and single-cell RNA sequencing to identify and validate underlying gene signatures from pre-treatment archived tumor specimens from patients enrolled in the IMvigor210 and CheckMate 275 ICI monotherapy clinical trials as well as information from TCGA UC dataset [44]. Bulk RNA sequencing data revealed a signature, the 2IR score, that is a ratio of this adaptive immune to protumorigenic inflammation. High 2IR was associated with better ICI outcomes. Single-cell RNA sequencing data was used to discover the diverse cellular populations associated with these gene signatures, and it revealed prominent expression of the protumorigenic inflammation signature by myeloid phagocytic cells. Patient tumor samples containing single myeloid phagocytic cells with low 2IR scores were associated with ICI-resistant aUC. These results highlight areas of interest for new therapies to target protumorigenic inflammation to potentially overcome ICI resistance.

In a retrospective study, 335 patients with aUC who received at least two cycles of pembrolizumab or atezolizumab from 2015-2023 were assessed to identify potential factors related to primary refractory disease, which was defined in the study as patients with clinical or radiologic progressive disease or death within the first 14 weeks of ICI initiation [45]. This cohort revealed that an elevated platelet to lymphocyte ratio ≥ 308, body mass index < 18.5, the use of antibiotics within 60 days after starting ICI, and upper tract disease were all associated with primary refractory disease. In another analysis of this cohort, there were 49 patients with pretreatment tissue biopsies available for NGS. Comparing 23 responders (complete response, partial response, and stable disease) to 26 non-responders (progressive disease), there were higher frequencies of *TP53*, *KMT2A*, *KMT2C*, and *ERBB2* alterations for patients who were responders [46].

With increasing knowledge about ICI resistance, trials are being designed to overcome this [47]. These efforts include evaluating antibodies against other immune checkpoints upregulated during immune exhaustion, such as a three-arm phase 2 trial (NCT05645692) of tobemstomig [a bispecific antibody against programmed cell death protein 1 (PD-1) and LAG-3] and tiragolumab (antibody against TIGIT) compared to tobemstomig alone or atezolizumab alone for patients with previously untreated aUC ineligible for PBC. Tumor vaccines to promote neoantigen presentation through various mechanisms, such as inoculation of neoantigen peptides, viruses, and mRNA vesicles, to stimulate the immune system are being evaluated in combination with ICIs [47, 48].

## Disruption of EVP synergy

The synergy between EVP has not been fully characterized, but some supporting evidence includes EV inducing an immunogenic cell death, EV upregulating major histocompatibility complex class I and II expression, and EV inhibiting nectin-4 from binding to TIGIT [49]. However, there are likely other mechanisms of resistance to EVP simultaneously that still need to be unraveled.

With no clear biomarkers of treatment response or resistance to EVP, it is imperative that patients are regularly followed up to monitor for clinical and radiographic signs of progressive disease. Additionally, serial quantitative circulating tumor DNA (ctDNA) assessment may have a role in the future. These assays can be tumor-informed or tumor agnostic, and preliminary studies have shown that a rise in ctDNA levels on treatment is likely suggestive of the development of treatment resistance and progressive disease on imaging [50]. Nonetheless, further prospective studies are necessary to determine their utility in clinical settings, including following response to EVP [51].

## Conclusions

EVP has been a breakthrough for patients with aUC. Resistance to EVP remains problematic, ultimately affecting the majority of patients, and these mechanisms are becoming better characterized. With deeper understanding of this resistance, as well as further knowledge gained from genetic and epigenetic studies, better predictive markers may be revealed, and improved therapies can be used to overcome the resistance.

Understanding resistance may also help guide subsequent therapies. For example, if the resistance to EVP is from downregulation of nectin-4, then the next line of treatment could be another ADC targeting a different surface marker but with the same cytotoxic payload, such as DV, which binds to HER2 but still uses MMAE. Alternatively, if resistance is mediated by a drug efflux pump, zelenectide pevedotin (BT8009), a bicyclic peptide much smaller than EV that interacts strongly with nectin-4 and is also conjugated to MMAE could overcome resistance. In contrast, if the resistance to EVP were to the MMAE agent, then a different toxin would have to be used, but the antibody against nectin-4 could remain the same.

Additionally, diverse targets and toxins may be used to overcome resistance to EVP. T-DXd is already FDA-approved as a tumor agnostic therapy for patients with HER2-positive (IHC 3+) expression [52]. The Double Antibody Drug Conjugate (DAD) phase 1 trial (NCT04724018) combined sacituzumab govitecan (SG), which binds to Trop2 and is conjugated to the toxin SN-38, and EV in patients with aUC previously treated with PBC or immunotherapy [53]. This trial demonstrated a high response rate of 70% for 23 patients, with only 3 patients having primary refractory disease. This trial will have another cohort of patients with previously untreated aUC to receive a triplet combination of SG, EV, and pembrolizumab (DAD-IO) [54]. The phase 3 trial DV-001 (NCT05911295) is evaluating the combination of DV and pembrolizumab for HER2-expressing previously untreated aUC. Despite EVP primary and secondary resistance, scientific breakthroughs and novel therapies hold promise to improve the outcomes for patients with aUC.

## Abbreviations

ADCs: antibody drug conjugates
aUC: advanced urothelial carcinoma
BV: brentuximab vedotin
CPS: combined positive score
ctDNA: circulating tumor DNA
DV: disitamab vedotin
EV: enfortumab vedotin
EVP: enfortumab vedotin and pembrolizumab
ICIs: immune checkpoint inhibitors
IHC: immunohistochemical
LAG-3: lymphocyte activation gene-3
MDR: multi-drug resistance
MMAE: monomethyl auristatin E
mOS: median overall survival
mPFS: median progression-free survival
NGS: next-generation sequencing
ORR: observed response rate
OS: overall survival
PBC: platinum-based chemotherapy
PD-1: programmed cell death protein 1
PD-L1: programmed cell death protein ligand 1
PFS: progression-free survival
SG: sacituzumab govitecan
T-DXd: trastuzumab deruxtecan
TIGIT: T cell immunoreceptor with immunoglobulin and ITIM domain
TIM-3: T-cell immunoglobulin and mucin domain-containing molecule 3
UNITE: Urothelial Cancer Network to Investigate Therapeutic Experiences

## Declarations

### Copyright

© The Author(s) 2025.
